# Direct measurement of the propagation of the phase-transition region of liquid crystals

**DOI:** 10.1038/srep44801

**Published:** 2017-03-20

**Authors:** Takahiro Sato, Kenji Katayama

**Affiliations:** 1 Department of Applied Chemistry, Chuo University, 1-13-27 Kasuga Bunkyo Tokyo, 112-8551, Japan; 2 JST, PRESTO, 4-1-8 Honcho, Kawaguchi, Saitama, 332-0012, Japan

## Abstract

Many types of active matter, such as biological cells, have liquid-crystalline membranes, which are soft and flexible in their interactions with their surroundings and sometimes allow molecular-structural or -orientational changes to extend for long distances, owing to long-range molecular interactions. Despite the technological and fundamental importance of these long-range changes, there is no good physical property with which to express them for the liquid crystal. Here, we show direct measurements of the propagation of structural or orientational changes due to long-range molecular interactions in liquid crystals. We induced a patterned phase transition in a liquid crystal via illumination with a fringe pattern and observed the propagation of the phase-transition region. We determined that the propagation occurred in a ballistic manner with a velocity of 80–110 m/s and that two types of propagation—side-by-side and head-to-tail molecular interactions—were found.

The structural or orientational change in a molecular assembly sometimes propagates in a collective manner owing to long-range molecular interactions, reaching distances on the order of millimetres. Such long-range change has been observed in the field of photoinduced phase transition for spin-crossover complexes^1^, conjugated polymers^2^ and strongly correlated electron systems interleaved by donor and acceptor layers^3^, which are caused by the macroscopically correlated interaction between electrons, spins and phonons. Other examples are found in liquid crystals (LCs), which can be encountered in nature in biological membranes, silks and the like. Unlike typical crystalline materials, such as metals or semiconductors, LCs feature softness, which enables them to possess curved structures similar to that of a cell membrane, keeping their periodic structure. An orientational or structural change of molecules in an assembly would trigger overall structural change by spatial expansion of the changed region.

Since LCs are soft, such interaction causes a ‘supple’ motion similar to that of leather, and they have been utilized frequently as a host material in active matter, which has been a recent area of interest for research. An active capsule composed of LCs was demonstrated, and the motion was controlled by a small number of defects, which means the position at which the direction of molecular orientation could not be defined^4^. A macroscopic flow was induced in LCs by injecting a small fraction of bacteria^5^. Micro-objects can be moved in an LC by controlling the solid-surface/liquid-crystal interaction^6^. In any active matter using LCs, a small molecular trigger induces the propagation of structural or orientational change owing to the molecular interaction of the LCs, causing a supple structural change. The key factors for this motion are how fast and for how long this change propagates.

Here, we came up with an idea to utilize the phase transition of LCs to understand how the structural or orientational change of molecules propagates in LCs because there should be an interface between the LC and isotropic phases during the phase transition. There was a study in which the interface of the phase change was imaged by applying a temperature gradient to an LC for the purpose of studying the mechanism of phase transition^7^. Here, we will measure the motion of the interface by a sophisticated time-resolved optical technique. A patterned light was illuminated onto an LC cell, in which guest dye molecules were put inside an LC for light absorption, causing the phase transition of the LC in a controlled way, and the propagation of the phase-transition region was measured by an optical technique known as transient grating (TG).

This type of optical excitation with a fringe pattern has been utilized for the study of the phase transitions^8^,^9^, reorientation by rotational relaxation^10^,^11^, optical nonlinearity^12^,^13^,^14^, acoustic or thermal properties^9^,^15^, and some applications such as holographic recording^16^,^17^ over the last 25 years. As host LCs, 4-methoxybenzylidene-4-n-butylaniline (MBBA) and p-pentyl-p’-cyanobiphenyl (5CB) have been frequently used because of their availability and their low temperature for the nematic phase. Depending on the guest molecules, various interesting phenomena were reported. By using azobenzene, the phase transition was induced at a much lower temperature than the phase-transition temperature from nematic to isotropic phase (T_NI_), which was called the photochemical phase transition^18^,^19^, and the mechanism was studied^20^,^21^. Using dyes such as azo dyes^22^,^23^,^24^ or anthraquinone dyes^25^, pre-transitional optical nonlinear response was observed close to the phase-transition temperature^26^. This was explained by the change in the anchoring condition as a result of the director reorientation^22^. Nanomaterials such as carbon nanotubes or quantum dots were used as guests, and optical properties were enhanced^27^,^28^. For these guest-induced effects, there are various explanations: guests work as defects, surface/guest interaction changes the orientation, the structural change of the guest induces the torque, etc.

Here, we utilized the TG technique again to realize the measurement of the motion of a phase-transitioned interface in a controlled fashion to study the molecular-interaction propagation for the first time. This study answers the fundamental question of how the structural or orientational changes of LCs propagate, and it leads to the understanding of the mechanism of the complicated host/guest interaction in LCs.

## Results

### Sample conditions

An LC sample doped with a small fraction of dye (2.7 mol%) was put into a thermally controlled LC cell in which the LC molecules were aligned in a specific direction. The polarization–absorption spectrum for each guest with 7OCB and 7CB in the LC cell was measured to confirm the absorbance at the pump wavelength (355 nm; see Supplementary Fig. S1). From the absorption differences for the parallel and perpendicular polarizations relative to the alignment direction, it was determined that the dipole of the guest molecules was mostly aligned along the director axis. The concentration was adjusted such that the absorbance was ~0.5 for the parallel configuration at the pump wavelength to ensure the uniform light absorption in the depth direction.

### Transient grating method and principle

**Figure 1:**
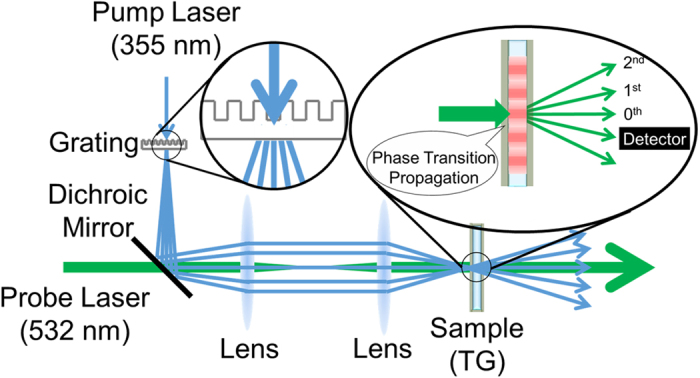
The setup for the transient-grating measurement.

To induce the patterned phase transition, we utilized the optical setup of the transient grating (TG) method. In typical TG techniques, two lights are crossed at the sample to generate a fringe pattern. Depending on the physical phenomena induced, various responses, such as thermal, acoustic or electrical responses, can be obtained from which various properties can be obtained^29^,^30^,^31^. We also developed a different optical setup to study the ordering/disordering or phase-transition dynamics of the liquid crystal^32^. However, in this study, another new optical setup was developed to irradiate at the same intensity even for different fringe spacings, as shown in Fig. 1. An excitation pulse was first passed through a glass slide having grating patterns with different grating fringes, and the pattern of the image was formed at the sample position using a 4 f configuration of two lenses with different focal lengths. The pump pulse was absorbed by the guest dyes, causing photoisomerization or release of heat. When the LC cell temperature was set close to the nematic–isotropic phase-transition temperature (T_NI_), the light absorption caused phase transition of the LC. Since the phase transition induced a change in the refractive index, the refractive-index pattern formed was the same as the pump-light pattern, which is called transient grating (TG). The TG was observed as the 1^st^-order diffraction intensity of a probe light, as measured with a photodiode. When the probe polarization was parallel to the director axis, the change in the extraordinary refractive index, Δn_e_, could be measured, whereas the ordinary index change, Δn_o_, could be observed for the probe polarization perpendicular to the director.

**Figure 2:**
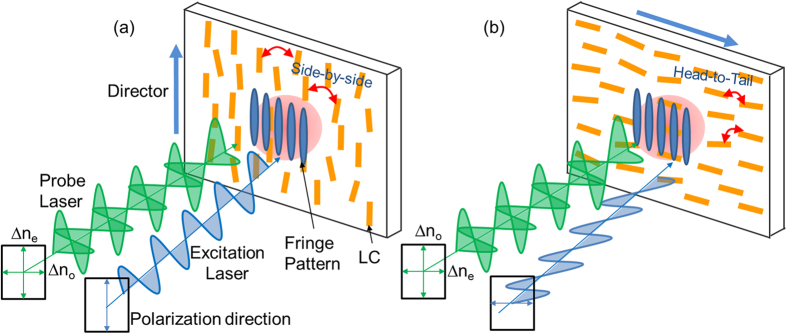
The sample configurations for the TG measurements. The pump fringe pattern is (**a**) parallel to the director and (**b**) perpendicular to the director. The polarization of the pump lights was always set parallel to the director axis. When the probe polarization was parallel or perpendicular to the director, Δn_e_ or Δn_o_ was measured.

We utilized two configurations of the sample and the polarizations of the pump and probe lights, as shown in Fig. 2. On the left side (a), the director was set parallel to the fringe pattern formed by the pump, whereas on the right side (b), it was set perpendicular to the fringe pattern. In either case, Δn_e_ was observed when the probe polarization was parallel to the director axis and Δn_o_ was detected for the probe polarization perpendicular to it.

The physical origin of the refractive-index change is divided into three components,


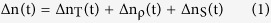


where the terms on the right side correspond to the index changes due to temperature, density and the order parameter. In previous studies of the dynamics of LCs, the Δn_T_(t) term was smaller than Δn_ρ_(t) and Δn_s_(t). The Δn_ρ_(t) term is caused by the density change due to the phase change^9^,^11^, while the Δn_s_(t) term is the ordering change of the LCs (i.e., the change in the order parameter). The last two components are supposed to be the signal origins of the TG responses in the phase-transition process^32^. The intensity of the TG signal is proportional to the square of Δn(t)^33^.

**Figure 3:**
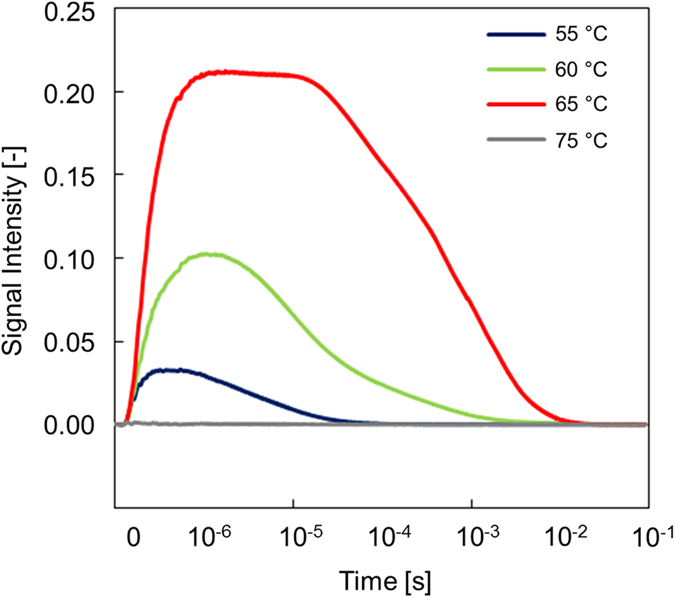
Initial-temperature dependence of the TG responses for 7OCB including azobenzene as a guest molecule. Configuration (a) shown in Fig. 2 was used.

First, to confirm that the phase transition of the LC was induced, the initial temperature dependence of the TG responses was investigated for configuration (a) in Fig. 2. The TG responses at Δn_e_ for 55, 60, 65 and 75 °C for Azo as a guest molecule are shown in Fig. 3. As shown in previous papers^32^,^34^, phase transitions were not induced for the initial temperature of 55 °C, which is much lower than the phase-transition temperature from the nematic to isotropic phase (T_NI_ = 74 °C), at which the host LCs were disordered by guest dyes and restored by reorientation processes such as ordering or rotation of the LCs. As the initial temperature increased, the Δn_e_ amplitude increased dramatically, and when it approached T_NI_, the Δn_e_ amplitude was enhanced seven- to eight-fold compared with that at 55 °C. It showed a plateau in the time region, followed by decay to the baseline on the order of milliseconds. This response corresponds to the phase transition and the following phase recovery of the host LCs. The phase transition was confirmed from the agreement between the values of Δn_e_ ≃0.1 calculated from the maximum diffraction efficiency, which in turn was obtained from the signal intensity in the saturated region, and the literature values of the refractive-index difference between the nematic and isotropic phases^35^,^36^. It was further confirmed by the fact that a minuscule change in the refractive index was observed once the initial temperature was set above T_NI_ because no phase change occurred under pump-light irradiation. (It should be noted that there is a chance that the observed phenomena correspond to the pretransitional phase transition, observed for the dye-doped LCs^24^, and this issue is not explored here in detail).

Note that the temperature difference between T_NI_ and the initial temperature did not agree with the pure temperature jump induced by the pump light because Azo induces both photothermal and photochemical effects in its phase transition owing to photoisomerization^37^,^38^. The pure temperature jump of the LC was obtained by the same experiments for p-nitrophenol (p-NP) as a guest molecule in the same host LC. Since p-NP only releases heat during the decay process, the difference between T_NI_ and the initial temperature when the phase transition began corresponds to the pure temperature jump, which was estimated to be 4 K.

We studied the phase-transition dynamics, namely, the rising part of Fig. 3. The dependence of the fringe spacing of the pump pattern was studied, and we found a systematic grating dependence in the phase-transition process only for the Δn_e_ responses, where the rise time increased as the fringe spacing increased. Since this rising part was caused by the formation of an isotropic region by irradiation with the pump light, the fringe-space dependence indicates that the isotropic region expanded spatially, namely, thickening the stripe region in Fig. 1. This is direct evidence that the phase-transition region propagated. (It is noted that the thermal grating, representing that the fringe-patterned temperature rise decays due to thermal diffusion, also depends on the fringe spacing, but the thermal grating decay occurs on the order of a few milliseconds depending on the fringe spacing for 5CB (thermal diffusivity ~6 × 10^−4^ cm^−2^), and the response is included in the decay part of Fig. 3.)

**Figure 4:**
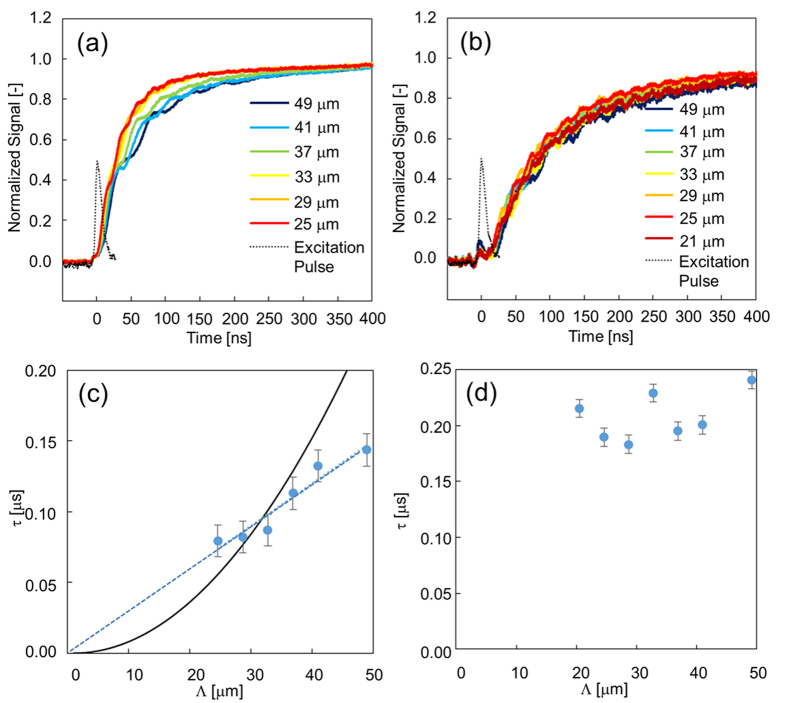
The TG responses for different fringe spacings in the initial rising parts of the responses. The setup configuration corresponds to that shown in Fig. 2(a). The sample was 7OCB, including azobenzene as a guest molecule. (**a**) and (**b**) correspond to Δn_e_ and Δn_o_, respectively. The measurement temperature was set at 65 °C. The timing of the pump-pulse irradiation is shown for each graph. The time constants for the rise component are shown for different fringe spacings with (**c**) and (**d**) corresponding to (**a**) and (**b**), respectively. Fitting curves for diffusive (black) and ballistic (blue-dotted) propagations are shown in (**c**).

Interestingly, this propagation was only observed for Δn_e_ and not for Δn_o_, as shown in Fig. 4. The refractive-index change for the LCs is primarily caused by changes in the density and ordering of molecules, as explained above^9^,^32^. Since the former should induce the same temporal response for Δn_e_ and Δn_o_, the difference between them is caused by the order-parameter change of the molecules. The nematic LCs are ordered only along the director axis, which is the origin of the extraordinary refractive index, and it is assumed that these ordered molecules were disordered and that the boundary between the ordered and the disordered regions travelled. This process should involve interactions between molecules in a side-by-side manner, possibly dipole/dipole interactions as shown in Fig. 2(a), thereby causing disordering. Conversely, the time constants for the responses in Fig. 4(b) had almost constant values of 200 ± 40 ns, corresponding to the density change owing to the phase change.

**Figure 5:**
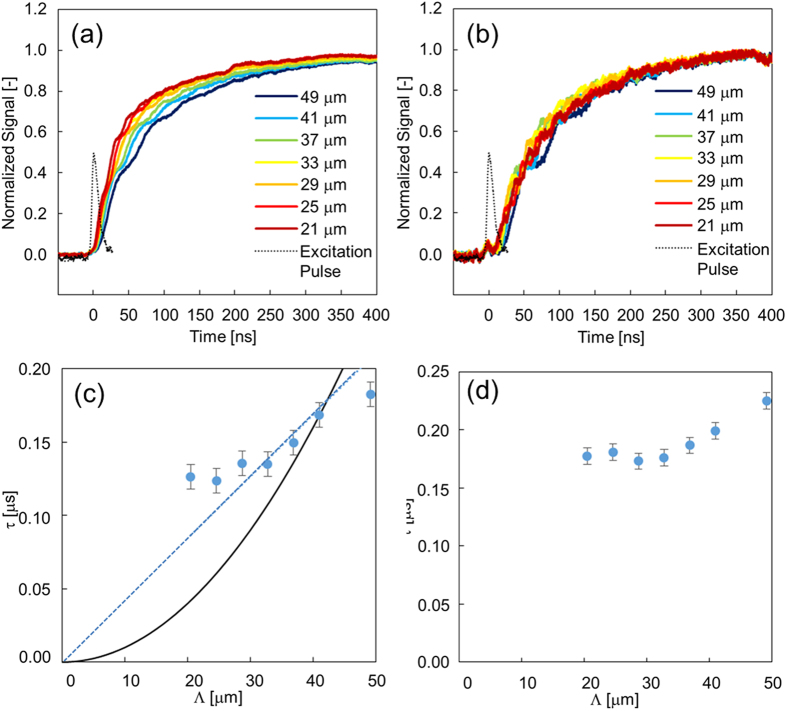
The TG responses for different fringe spacings in the initial rising parts of the responses. The setup configuration corresponds to Fig. 2(b). The sample was 7OCB including azobenzene as a guest molecule. (**a**) and (**b**) correspond to Δn_e_ and Δn_o_, respectively. The measurement temperature was set to 65 °C. The timing of the pump pulse irradiation is shown for each graph. The time constants for the rise component are shown for different fringe spacings in (**c**) and (**d**), corresponding to (**a**) and (**b**), respectively. Fitting curves for diffusive (black) and ballistic (blue-dotted) propagations are shown in (**c**).

To see if this propagation of structural/orientational disorder occurs in the other direction, similar measurements were conducted for the configuration of Fig. 2(b), namely, with the fringe perpendicular to the director axis. The fringe-spacing dependence was still observed only for the Δn_e_ response (Fig. 5). Thus, even for a different excitation pattern for the LC, only the region caused by Δn_e_ could propagate. This indicates that the molecules could interact with other molecules in a head-to-tail manner, as shown in Fig. 2(b). The time constants for the Δn_o_ responses in Fig. 5(b) showed almost constant values: 200 ± 30 ns, corresponding to the density change due to the phase change. Since this value agreed with that for Fig. 5(b), this result supports the conclusion that the time constants for Δn_o_ correspond to the density change of the phase transition.

To confirm that these phenomena are general for other host LCs, the same observations as in Figs 4 and 5 were made for 7CB as a host LC. The TG responses and the same analyses are shown in Supplementary Figs S2 and S3 for the configurations of Fig. 2(a) and (b), respectively. The general tendency was almost the same as that in the 7OCB/AZO case. The fringe-spacing dependences were observed only for the Δn_e_ responses, suggesting that the disordering of the molecule in the director direction could expand for 7CB as well. The difference was that the density changes due to the phase transition observed in Δn_o_ were a little longer than for 7OCB, namely 250 ± 50 ns. Additionally, we report that this propagation was observed for a different guest molecule, p-nitrophenol, as shown in Supplementary Fig. S4.

Furthermore, the type of molecular-interaction propagation was studied. As a simple consideration, propagations can be categorized into two types: diffusive and ballistic. The former indicates that the propagation occurs with scattering events, while the latter indicates a constant propagation speed without scattering.

To analyse the TG response due to the phase transition, the square roots of the normalized TG responses were calculated and fitted with a single exponential function: 1 − exp(−t/τ) (τ: time constant). When the time constant is dependent on the fringe spacing of the pump pattern, it indicates that the phase-transition region propagates; this means that the isotropic region (stripe pattern) in Fig. 1 thickens. The time constant is given when the propagation is diffusive,


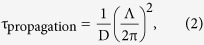


where D is the diffusion coefficient and Λ is the fringe spacing. When the propagation is ballistic,


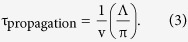


Here, v is the velocity of phase-transition propagation.,

The time constant is proportional to the square of the fringe spacing for diffusive propagation, whereas it is proportional to the fringe spacing for ballistic propagation. The graphs of the time constant versus the fringe spacing are shown in Figs 4,5, S2 and S3 for both Δn_e_ and Δn_o_. The graphs show that this propagation occurred in a ballistic manner for side-by-side propagation, and some deviation was observed for the head-to-tail propagation, possibly because the diffusive mechanism was mixed. From the slopes of each graph, the propagation speeds were estimated. For 7OCB, the side-by-side interaction was 110 m/s, and the head-to-tail interaction was 80 m/s. For 7CB, they were 110 m/s and 110 m/s, respectively. The propagation speeds were approximately one-tenth of the acoustic velocity, which can be noticed from comparison with the acoustic responses described later. The speed difference between the side-by-side and the head-to-tail interactions could be explained by the molecular structure, but the physical and chemical origin is still unclear, and further theoretical investigations are necessary, such as a simulation of the molecular dynamics, which we plan to do in the near future.

In the rising part of the responses, the oscillations overlapped. This part corresponds to the acoustic signal of the LC in this case, which is typically observed by the general TG measurements^39^. Owing to thermal expansion by light absorption, a standing wave was generated with a wavelength equal to the fringe spacing. This acoustic wave induced the density modulation, causing the refractive-index modulation, inside the sample, which works as a transient grating. The acoustic-wave velocity could be estimated from Λ/T_interval_ (Λ: fringe spacing; T_interval_: period of the acoustic oscillations). The acoustic velocities were 1,250 ± 70 m/s and 1,350 ± 70 m/s for 7OCB and 7CB, respectively. These values are somewhat different from those in the literature (1.7 × 10^3^ for 7CB at 300 K)^40^. Since the observed acoustic wave was the one exactly in the middle of the phase transition, the acoustic velocities cannot be compared with the literature values obtained under a static condition. Because this issue is beyond the scope of this paper, it will be dealt with in the future.

In conclusion, we could successfully observe the propagation of the structural or orientational change of LCs in the phase transition from the nematic to isotropic phase induced by the light absorption of the guest molecules. This propagation was only observed in the direction of the director axis, and the propagation occurs in a ballistic manner with a speed of approximately 100 m/s. Since many types of host/guest molecular interactions have been proposed for optical applications, this method opens up the study of the characteristic properties of the host/guest system of LCs.

## Method

An Nd:YAG pulse laser with a wavelength of 355 nm, a pulse width of 5 ns and an intensity of 0.5 mJ/pulse (GAIA, Rayture systems) was used as a pump source. Glass with different fringe patterns (spacing: 20–100 μm) was used as a target pattern at the sample position. The pattern was formed using a 4 f configuration of two lenses with different focal lengths: 80 and 33 mm. The magnification of the pattern was 61%. An Nd:YVO_4_ laser with wavelength 532 nm and intensity 1 mW (JUNO, Showa Optronics) was used as a probe source. The detector was a photodiode with a fast response (DET110, Thorlabs), and this response was amplified with a voltage amplifier (DHPVA, Femto). The voltage response was observed by an oscilloscope (Wave Runner 6100 A, Lecroy).

We measured two configurations of the sample, the pump and the probe lights, as shown in Fig. 2. In (a), the director was set parallel to the fringe pattern formed by the pump, whereas it was set perpendicular to it in (b). In both cases, Δn_e_ was observed when the probe polarization was parallel to the director axis, and Δn_o_ was detected with the probe polarization perpendicular to it.

4-Cyano-4′-n-heptyloxybiphenyl (7OCB) and 4-Cyano-4′-n-heptylbiphenyl (7CB) were used as host LCs. The temperature ranges for the LC state were 54–74, and 25–42 °C, respectively. Azobenzene (AZO) and p-nitrophenol (p-NP) were used as photochemical and photothermal guest molecules, respectively. With the addition of guest molecules, T_NI_ was approximately 2 °C lower than that for pure LCs. All chemicals were purchased from Tokyo Kasei and used without further purification. The sample was put into an LC cell (E.H.C Corp.) with a sample thickness of approximately 3 ± 0.5 μm with rubbed polyimide films inside. The LC cell was covered by an aluminium frame whose temperature was controlled by a heater controller (TC200, Thorlabs). The initial temperatures for 7OCB and 7CB were set at 65 and 35 °C, respectively. An LC sample doped with a small fraction of a guest (2.7 mol%) was put into a thermally controlled LC cell, where the LC molecules were aligned in a specific direction. The dye concentration was adjusted such that the absorbance of the samples corresponded to 0.5.

## Additional Information

**How to cite this article**: Sato, T. and Katayama, K. Direct measurement of the propagation of the phase-transition region of liquid crystals. *Sci. Rep.* **7**, 44801; doi: 10.1038/srep44801 (2017).

**Publisher's note:** Springer Nature remains neutral with regard to jurisdictional claims in published maps and institutional affiliations.

## Supplementary Material

Supplementary Information
