# Supplementary material for: Direct measurement of the propagation of the phase-transition region of liquid crystals
Source: Sci Rep. 2017 Mar 20;7:44801. doi: 10.1038/srep44801 (PMC5357855; doi:10.1038/srep44801)
Supplement: Supplementary Information [file srep44801-s1.pdf]

Supplementary Information

Direct measurement of the propagation the phase-transition region of liquid crystals

Takahiro Sato[1] and Kenji Katayama[1,2]*

[1] Department of Applied Chemistry, Chuo University, 1-13-27 Kasuga Bunkyo Tokyo, Japan, 112-8551

[2] JST, PRESTO, 4-1-8 Honcho, Kawaguchi, Saitama, 332-0012, Japan

*Author for correspondence: Kenji Katayama, PhD

Department of Applied Chemistry, Chuo University, 1-13-27 Kasuga Bunkyo Tokyo, Japan, 112-8551

Tel: +81-3-3817-1913, E-mail: kkata@kc.chuo-u.ac.jp

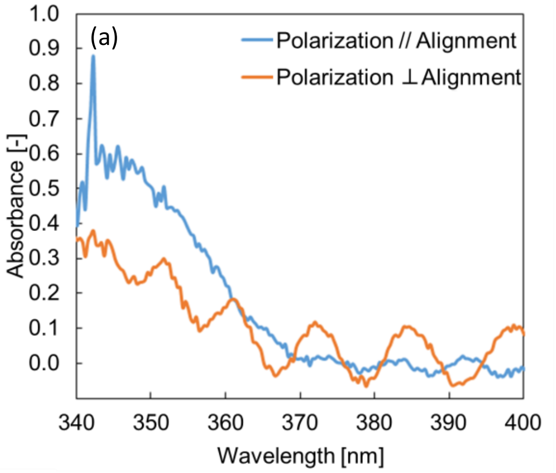
(a)
Polarization // Alignment
Polarization ⊥ Alignment
Absorbance [-]
1.0
0.9
0.8
0.7
0.6
0.5
0.4
0.3
0.2
0.1
0.0
340
350
360
370
380
390
400
Wavelength [nm]

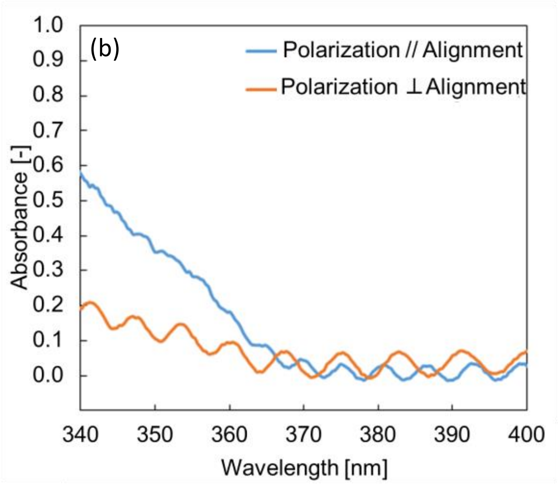
(b)
Polarization // Alignment
Polarization ⊥ Alignment
Absorbance [-]
1.0
0.9
0.8
0.7
0.6
0.5
0.4
0.3
0.2
0.1
0.0
340
350
360
370
380
390
400
Wavelength [nm]

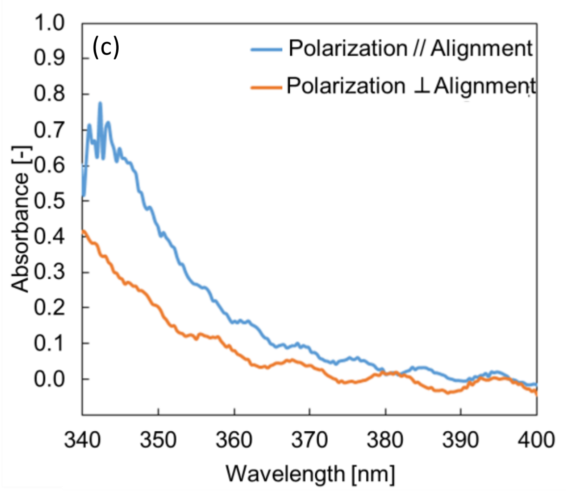
(c)
Polarization // Alignment
Polarization ⊥ Alignment
Absorbance [-]
1.0
0.9
0.8
0.7
0.6
0.5
0.4
0.3
0.2
0.1
0.0
340
350
360
370
380
390
400
Wavelength [nm]

Figure S1. Polarization absorption spectra for (a) azobenzene in 7OCB and (b) azobenzene in 7CB and (c) p-nitrotphenol in 7OCB in the liquid crystal cell. The polarization was set parallel and perpendicular to the director. The oscillation was observed due to the light interference in a thin LC cell. (3 μm in thickness)

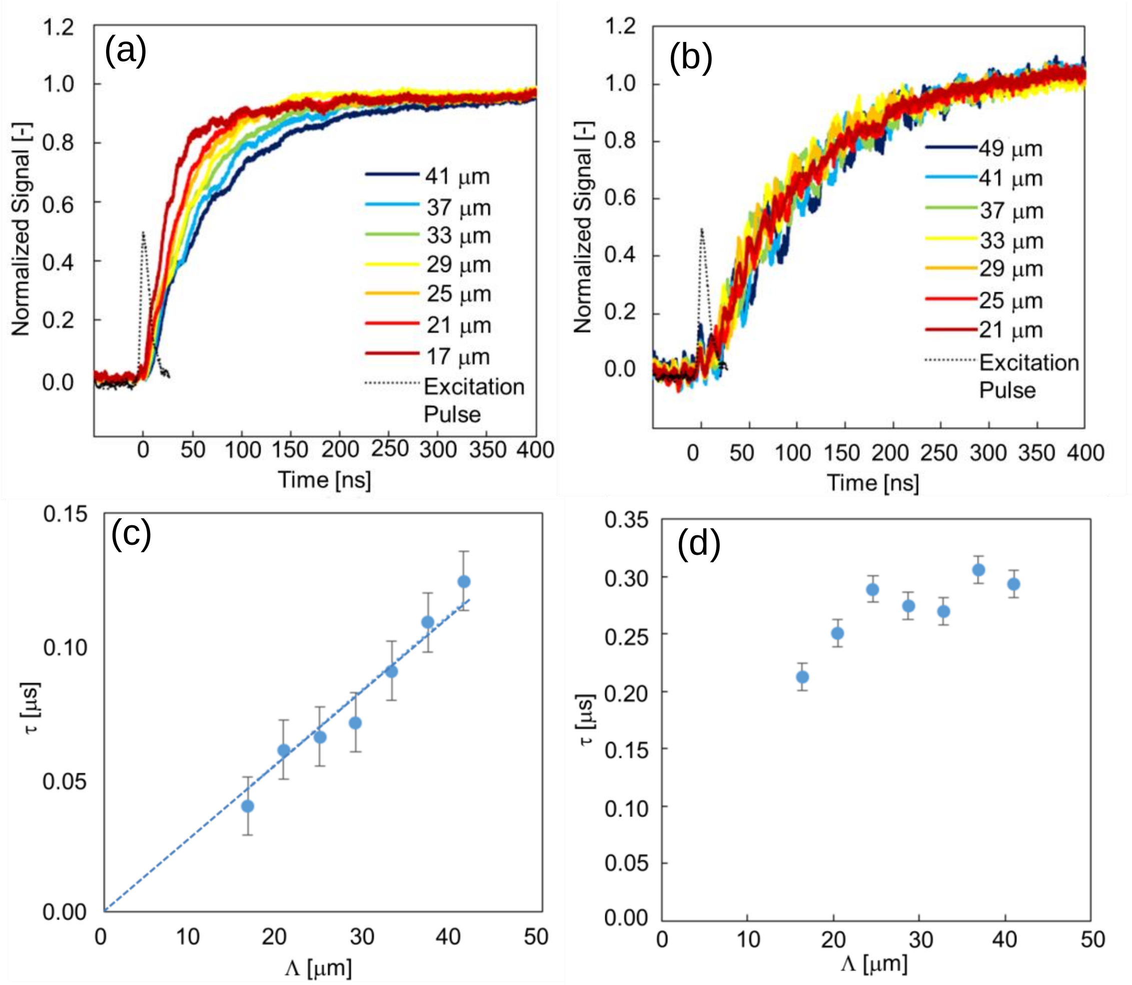

Figure S2. The TG responses for different fringe spacing in the initial rising part of the responses. The setup configuration corresponds to Fig.2(b). The sample was 7CB including azobenzene as a guest molecule. (a) and (b) correspond to Δn_e and Δn_o, respectively. The measurement temperature was set at 25 ^{o}C. The timing of the pump pulse irradiation was shown for each graph. The time constants for the rise component were shown for the different fringe spacing, (c) and (d), corresponding to (a) and (b), respectively. Fitting curves for diffusive or ballistic propagations are shown in (c).

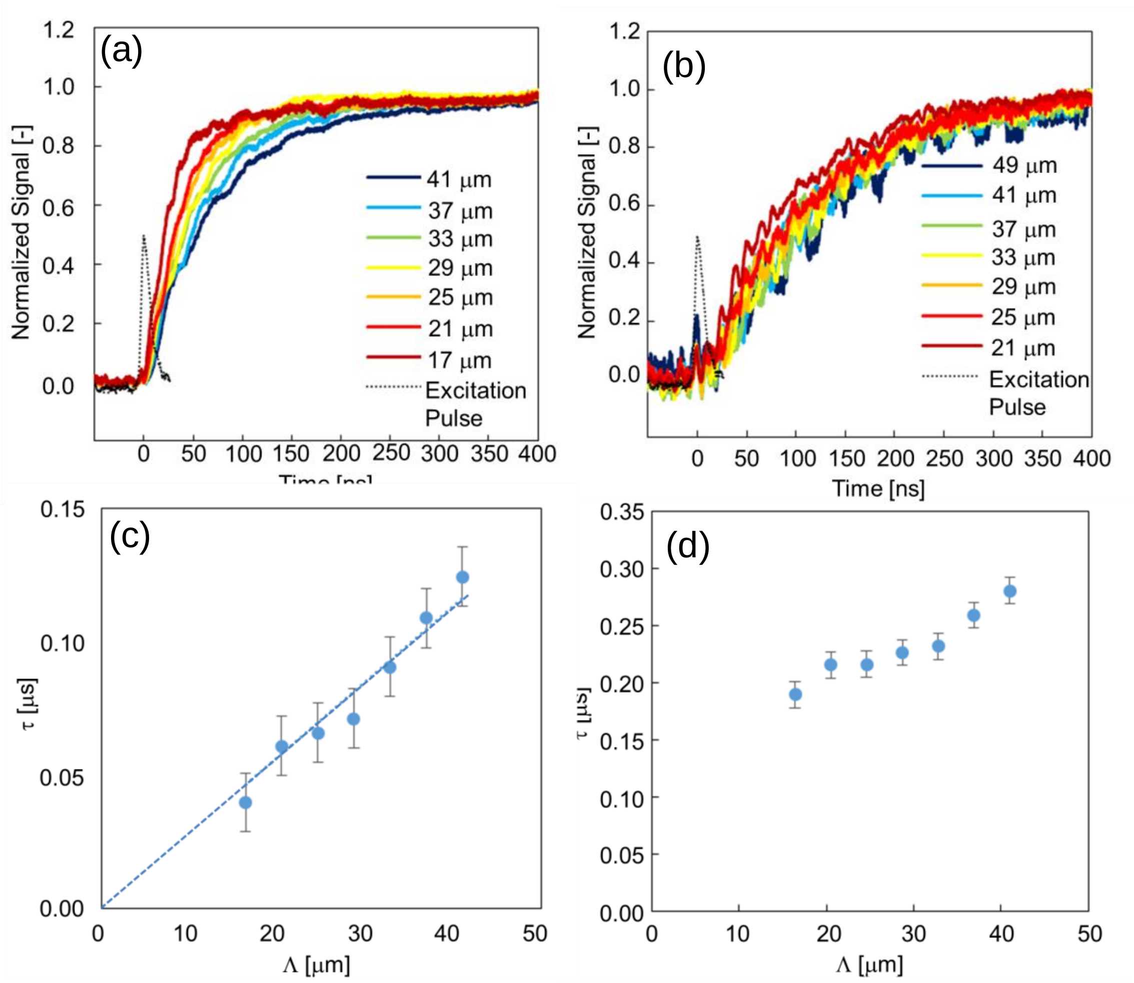

Figure S3. The TG responses for different fringe spacing in the initial rising part of the responses. The setup configuration corresponds to Fig.2(b). The sample was 7CB including azobenzene as a guest molecule. (a) and (b) correspond to Δn_e and Δn_o, respectively. The measurement temperature was set at 25 ^{o}C. The timing of the pump pulse irradiation was shown for each graph. The time constants for the rise component were shown for the different fringe spacing, (c) and (d), corresponding to (a) and (b), respectively. Fitting curves for diffusive or ballistic propagations are shown in (c).

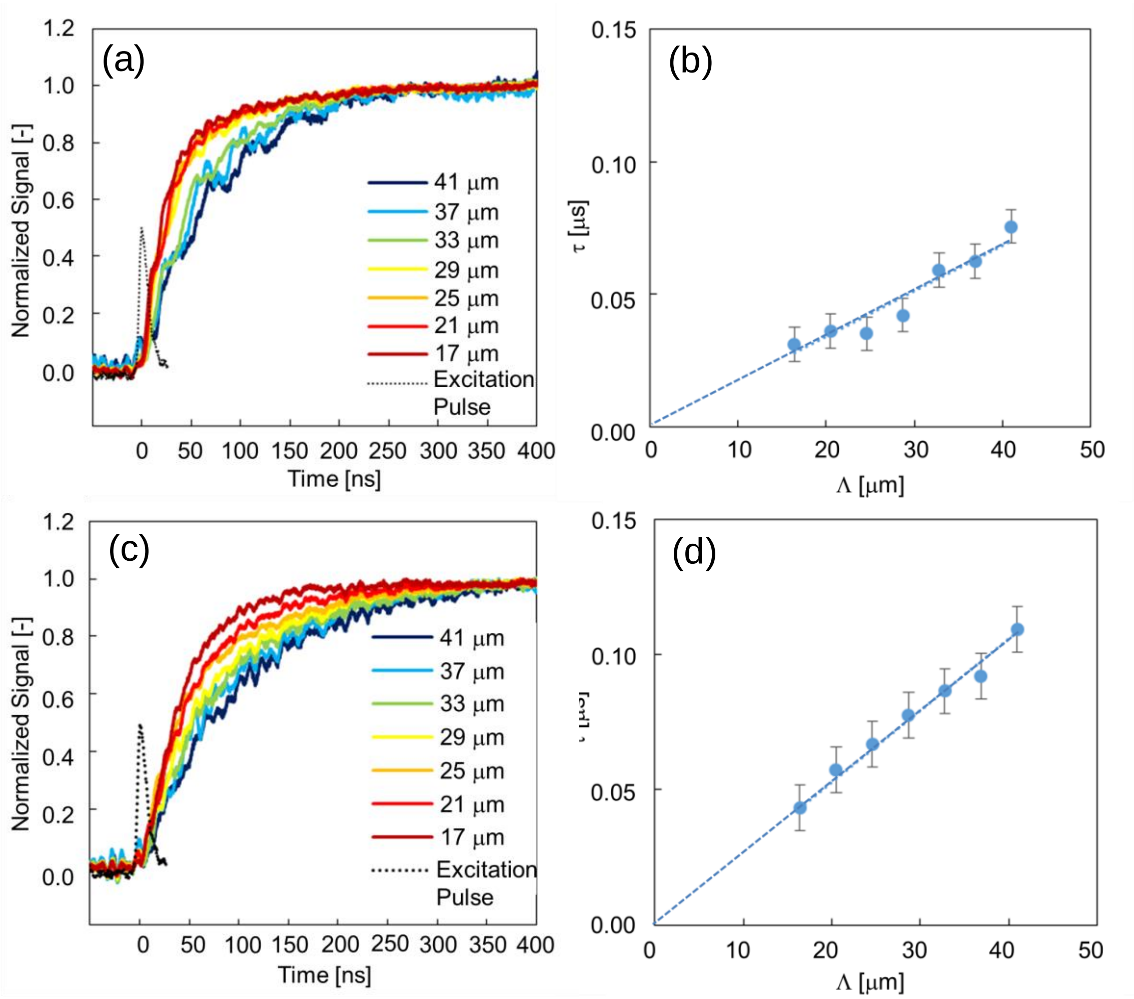

Figure S4. The TG responses for different fringe spacing in the initial rising part of the responses. The setup configuration corresponds to Fig.1(a) for (a) and (b), and Fig.1(b) for (c) and (d). The sample was 7OCB including azobenzene as a guest molecule. The responses of (a) and (c) correspond to Dne. The measurement temperature was set at 65 oC. The timing of the pump pulse irradiation was shown for each graph. The time constants for the rise component were shown for the different fringe spacing, (b) and (d), corresponding to (a) and (c), respectively. Fitting curves for diffusive are shown.
